# Morning Side

**Published:** 1870-02

**Authors:** 


### MORNING SIDE.

On a beautiful September day, we once found ourselves on the way to "Morning Side," then the residence of the world-famed Dr. Thomas Chalmers, and we have very pleasant memories of the interview with the great man, his wife, and daughters. What an odd name it was! we can't get at the idea of any morning having a "side" to it; but we can get at the meaning of

SUNNY SIDE.

There is the sunny side of Broadway; the sunny side of human nature; and the sunny side of the street: then again, there is the sunny side of a house; it is always the most healthy side. A sleeping apartment should be always on the sunny or southern side, for then it will be dry, and warm, and cheerful; there is superabundance of life in it. The prisoner, as he was marching to execution from a dark dungeon, in which he had been kept uninterruptedly for many years, as the morning sun shone in all his splendor, exclaimed: "What a beautiful world this is." John Brown is said to have made a similar exclamation, as he gazed on the lovely hills of old Virginia from his gallows stand. Then again, there is the sunny side of a man's heart, that always shines on his brother man, the evil and the good of them; always ready to do him a kindness, whether it is appreciated or not. Ah, that is God-like! such a man will be an angel in heaven one day. The Harland Pages, the Peter Coopers, the Lenoxes, the Peabodys, and Mills, and Nettleton, and Cornelius, and Judson, and the long list of self-denying, sainted foreign missionaries, and the home missionaries too, the Whitefields, and the Wesleys, and others like them who worked and preached in poverty, in buffetings and imprisonments, and still for all that maintained a sunny side in their hearts toward the very men who stoned them. Reader, have you a sunny side in your heart that never sets? then it will not be for long before the master will take you by the hand, and say, "Brother, come up higher."—*Ed. J. H.*

